# Comparison of seven in silico tools for evaluating of daphnia and fish acute toxicity: case study on Chinese Priority Controlled Chemicals and new chemicals

**DOI:** 10.1186/s12859-020-03903-w

**Published:** 2021-03-24

**Authors:** Linjun Zhou, Deling Fan, Wei Yin, Wen Gu, Zhen Wang, Jining Liu, Yanhua Xu, Lili Shi, Mingqing Liu, Guixiang Ji

**Affiliations:** 1 Nanjing Tech University, Nanjing, 211816 China; 2 Nanjing Institute of Environmental Sciences, Ministry of Ecology and Environment, Nanjing, 210042 China

**Keywords:** QSAR, Category, Aquatic toxicity, Daphnia, Fish, In silico

## Abstract

**Background:**

A number of predictive models for aquatic toxicity are available, however, the accuracy and extent of easy to use of these in silico tools in risk assessment still need further studied. This study evaluated the performance of seven in silico tools to daphnia and fish: ECOSAR, T.E.S.T., Danish QSAR Database, VEGA, KATE, Read Across and Trent Analysis. 37 Priority Controlled Chemicals in China (PCCs) and 92 New Chemicals (NCs) were used as validation dataset.

**Results:**

In the quantitative evaluation to PCCs with the criteria of 10-fold difference between experimental value and estimated value, the accuracies of VEGA is the highest among all of the models, both in prediction of daphnia and fish acute toxicity, with accuracies of 100% and 90% after considering AD, respectively. The performance of KATE, ECOSAR and T.E.S.T. is similar, with accuracies are slightly lower than VEGA. The accuracy of Danish Q.D. is the lowest among the above tools with which QSAR is the main mechanism. The performance of Read Across and Trent Analysis is lowest among all of the tested in silico tools. The predictive ability of models to NCs was lower than that of PCCs possibly because never appeared in training set of the models, and ECOSAR perform best than other in silico tools.

**Conclusion:**

QSAR based in silico tools had the greater prediction accuracy than category approach (Read Across and Trent Analysis) in predicting the acute toxicity of daphnia and fish. Category approach (Read Across and Trent Analysis) requires expert knowledge to be utilized effectively. ECOSAR performs well in both PCCs and NCs, and the application shoud be promoted in both risk assessment and priority activities. We suggest that distribution of multiple data and water solubility should be considered when developing in silico models. Both more intelligent in silico tools and testing are necessary to identify hazards of Chemicals.

## Background

Global regulations have called for systematic testing of potential environmental contaminants to protect human health and the environment from exposure to anthropogenic chemicals, such as industrial chemicals and pharmaceuticals. Considering the ever-increasing number of chemicals, more than 350,000 chemicals and mixtures of chemicals been registered for production and use currently [1], are presenting challenges to traditional ecotoxicity testing strategies for in vivo experiments, which are expensive, time-consuming, and reliant on large number of animal subjects. Therefore, it is virtually impossible to test acute toxicity for all the chemicals used globally.

To mitigate the challenges associated with in vitro and in vivo toxicity testing, global regulations, including European Chemical Agency (ECHA) REACH initiative, U.S. Toxic Substances Control Act and Canadian Environmental Protection Act, encourage increased reliance on in silico approaches [2–5]. China is also attempting to explore the possibility using in silico approaches when chemicals risk assessment.

The cost-benefit advantages and regulatory support of in silico methods have led to the development of a number of tools for ecotoxicity assessments [6]. The major in silico methods including (Quantitative) Structure–Activity Relationships (QSAR), and chemical category methods.

QSAR method uses a mathematical model that was derived from a training set of example chemicals. The training set includes the chemicals that were found to be positive and negative in a given toxicological study (e.g., the bacterial reverse mutation assay) or to induce a continuous response (e.g., Lowest Observed Adverse Effect Level in teratogenicity) that the model will predict. As part of the process to generate the model, physicochemical property based descriptors (e.g., molecular weight, octanol water partition coefficient (*K*_ow_)), electronic and topological descriptors (e.g., quantum mechanics calculations), or chemical structure-based descriptors (e.g., the presence or absence of different functional groups) are generated and used to describe the training set compounds. The model encodes the relationship between these descriptors and the (toxicological) response. After the model is built and validated, it can be used to make a prediction. The (physical) chemical descriptors incorporated into the model are then generated for the test compound and are used by the model to generate a prediction. This prediction is only accepted when the test compound is sufficiently similar to the training set compounds (i.e., it is considered within the applicability domain of the QSAR model, often considering the significance of descriptors). This applicability domain analysis may be performed automatically by some software to determine whether the training set compounds share similar chemical and/or biological properties with the test chemical [7].

Chemicals whose physical-chemical, toxicological and ecotoxicological properties are likely to be similar or follow a regular pattern as a result of structural similarity may be considered as a group, or ‘category’ of chemicals. The assessment of chemicals by using this category approach differs from the approach of assessing them on an individual basis, since the properties of the individual chemicals within a category are assessed on the basis of the evaluation of the category as a whole, rather than based on measured data for any one particular chemical alone. For (a) category member(s) that lacks data for one or more endpoints, the data gap can be filled in a number of ways, including by read-across from one or more other category members. Within a chemical category, the members are often related by a trend in an effect for a given endpoint, and a trend analysis can be carried out through deriving a model based on the data for the members of the category [8].

In 2007, the Organization for Economic Co-operation and Development (OECD) guidelines on the development and validation of QSAR models were issued [9]. They proposed that a QSAR model for practical application should be associated with an unambiguous algorithm [10], a defined endpoint, an AD, appropriate goodness-of-fit measures, robustness as well as predictive ability, and a mechanistic interpretation, if possible [9, 11]. Despite these guidelines, lack of external validations and model performances of the test sets, model overfitting, and poor AD definitions remain major concerns [12–15]. A clear AD definition would ensure that the model assumptions are met [16, 17].

A number of studies developed in silico models for the endpoint of acute toxicity to daphnia and fish [18–22]. Specifically, some in silico tools were developed for ecological risk assessment and are widely used for support chemicals regulation purpose. These include: Ecological Structure Activity Relationships (ECOSAR) [23], Toxicity Estimation Software Tool (T.E.S.T.) [24], Kashinhou Tool for Ecotoxicity (KATE) [25], Virtual models for property Evaluation of chemicals within a Global Architecture (VEGA) [26], Danish QSAR Database (Danish Q.D.) [27], and QSAR Toolbox developed by OECD [28].

In view of the possible uses of in silico tools, regulators often use predictions from multiple in silico tools to arrive at a decision, such as persistence, bioaccumulation,and toxicity/very persistent and very bioaccumulative (PBT/vPvB) assessment and prioritization [29]. In framework of regulation purpose, the performance of in silico tools requires not only accuracy, but also ease of use, and can fulfil the different purpose, such as qualitative risk assessment, quantitative risk assessment, and even high throughput screening [30].

Based on models for specific chemical classes and different classes of substances, some studies have compared the performance of some QSAR models for acute toxicity. Moore et al. [31] evaluated model performance of six QSAR modeling packages that predict acute toxicity to fish: ECOSAR, TOPKAT, a Probabilistic Neural Network, a Computational Neural Network, the QSAR components of the Assessment Tools for the Evaluation of Risk (ASTER) system, and the Optimized Approach Based on Structural Indices Set (OASIS) system. Golbamaki et al. [32] evaluated and compared eight in silico modelling packages that predict daphnia acute toxicity: TOPKAT, ACD/Tox Suite, ADMET Predictor™, ECOSAR, TerraQSAR™, T.E.S.T. and two models implemented in VEGA. Cassotti et.al [33]. evaluated the accuracy, stability and reliability of two acute toxicity models (MICHEM and ChemProp) to daphnia.

However, some of those evaluated tools were not easy to use and were not developed for regulatory purposes. These evaluation study did not include recently developed models, such as QSAR Toolbox, Danish Q. D., KATE, or the latest version of prediction tools, such as VEGA. Finally, the performance of chemical category approach for predicting acute toxicity to fish and daphnia has not been evaluated.

To implement the regulatory requirements of the “Action Plan for Prevention and Control of Water Pollution,” the Ministry of Ecological Environment of China issued the List of Priority Controlled Chemicals (PCCs) (the first batch) at the end of 2017 [34]. List of PCCs (the second batch) has been compiled and is under comment [35]. Most of these PCCs had been assessed shown the characteristic of PBT/vPvB, especially hazard to aquatic ecosystem. If a model can identify such eventually hazard-determining chemicals, it has great regulation application prospects. In addition, in silico tools should also be able to predict the hazard of emerging chemical substances in order to respond to the premanufacture notification for new chemical substances.

In this study, we selected seven in silico tools, namely ECOSAR, T.E.S.T., Danish Q. D., VEGA, KATE, Read Across and Trent Analysis, to predict acute aquatic toxicity to daphnia and fish, in order to provide insight into the applicability, accuracy and ease of use (convenience and the level of expert knowledge required) of these in silico tools. The testsets used in this evaluation were PCCs which are representative the final chemicals in the regulatory management process and NCs which are representative of emerging substances.

## Methods

### Validation datasets

Systematic and rigorous model evaluation requires reliable experimental data. As such, acute aquatic toxicity experimental data (48-h LC_50_ for daphnia and 96-h LC_50_ for fish) of PCCs with a great reliability were obtained from resources such as ECHA’s risk assessment report, Good Laboratory Practice (GLP) reports, or study with standard test methods were prioritize used. Other sources, such as ECHA, OECD eChemPortal database and QSAR Toolbox were also considered. If more than one data existed, a lowest reasonable value was used. Daphnia species were consist of *Daphnia magna*, *Daphnia pulex*. Fish species were consist of *Lepomis macrochirus*, *Cyprinus carpio*, *Pimephales promelas*, *Poecilia reticulate*, *Oncorhynchus mykiss*, *Oryzias latipes*, and *Brachydanio rerio et.al.* within *Actinopterygii*.

A total of 92 NCs tested were used after removing the mixture and UVCBs (Chemical Substances of Unknown or Variable Composition, Complex Reaction Products and Biological Materials), within which, there are 42 daphnia 48-h LC_50_ value and 82 fish 96-h LC_50_ value. These NCs were tested at the year from 2014 to 2017 using OECD testing guideline 202 [36] and 203 [36] under the GLP conditions in Lab of Chemical Testing and Assessment, Nanjing Institute of Environment Sciences, Ministry of Environment Protection (MEP), China. Daphnia species were *Daphnia magna*, and fish species were zebra fish*.* As these NCs came from chemical companies, the testing data is used for registration as the requirement of Measures for Environmental Management of New Chemical Substances in China. For confidentiality requirements, identification information of these NCs such as structural can not be provided. The functional groups contained were used to analysis and were obtained by module of organic functional groups (nested) in QSAR Toolbox.

### Predictive tools

The following seven in silico methods were evaluated for predicting acute aquatic toxicity to daphnia and fish: ECOSAR, T.E.S.T., Danish Q. D., VEGA, KATE, Read Across in QSAR Toolbox, and Trent Analysis in QSAR Toolbox. All of seven in silico tools were evaluated with PCCs dataset. Five tools including ECOSAR, T.E.S.T., Danish Q. D., VEGA and KATE were evaluated with NCs dataset.

Simplified Molecular Input Line Entry System (SIMLES) of each chemicals was used as input to models. A brief description of each program is provided below, and the pertinent details are summarized in Table 1.

**Table 1:** Summary of the predictive tools

Parameters	ECOSAR	T.E.S.T.	Danish Q.D.	VEGA	KATE	Read Across	Trent Analysis
Version	V2.0	V4.2.1	Not available	V1.1.5	2020 V1.0	QSARToolbox V4.4	QSARToolbox V4.4
AD definition	*K*_OW_ range	Structural similarity	Not available	Five AD index	*K*_OW_ range	Kow range and functional groups similarity	Kow range and functional groups similarity
AD judge	Manually	Automatic	Automatic	Automatic	Automatic	Automatic	Automatic
Algorithm	QSAR, Linear regression	QSAR, Hierarchical clustering, Single model, Group contribution, FDA, Nearest neighbor	QSAR, Leadscope: partial least squares, partial logistic regression; SciQSAR: regression on principal components and partial least squares regression, et.al.	Seven models for fish, covering QSAR and Read Across; two QSAR models for *Daphnia magna*,	QSAR, Linear regression	Chemical Category	Chemical Category
Daphnia	*Daphnia magna*, *Daphnia pulex*	*Daphnia magna*	*Daphnia magna*	*Daphnia magna*	*Daphnia magna*	*Daphnia magna*	*Daphnia magna*
Training sets size of daphnia	1000	353	626	Not available	562	> 1	> 1
Fish species	*Lepomis macrochirus*, *Cyprinus carpio*, *Pimephales promelas*, *Poecilia reticulate*, *Oncorhynchus mykiss*, *Oryzias latipes*, and *Brachydanio rerio*	*Pimephales promelas*	*Pimephales promelas*	*Pimephales promelas*, et.al.	*Oryzias latipes*, *Pimephales promelas*	*Actinopterygii*	*Actinopterygii*
Training sets size of fish	1000	823	565	Not available	535	> 1	> 1

#### ECOSAR

ECOSAR estimates acute aquatic toxicity via the Mayer–Overton relationship for chemicals within structurally similar classes. ECOSAR is trained on a large data set of ecotoxicity studies from the ECOTOX database that follow the U.S. EPA Office of Chemical Safety and Pollution Prevention guidelines, which comprise 130 structural classes. The log10 *K*_OW_ values for each training set chemical is predicted using the KOWWIN program from U.S. EPA’s Estimation Programs Interface Suite (EPISuit) model. The linear regression models between the LC_50_ toxicity estimates and log10 *K*_OW_ were developed for substances in each class. The predicted results of acute toxicity of fresh water other than saltwater were select to validation. Chemicals that do not meet the log10 *K*_OW_ range are considered to lie outside the AD.

#### KATE

KATE estimates acute aquatic toxicity via the Mayer–Overton relationship for chemicals within a total of 40 structural chemical classes [37, 38]. KATE is trained on the US EPA fathead minnow (*Pimephales promelas*) and the Japanese Ministry of Environment *Oryzias latipes* datasets [25]. The log *K*_*OW*_ value of the test chemical, which is obtained from an internal experimental database or is estimated with the alternative forced choice method. The relationship between LC_50_ value and log10 *K*_ow_ is obtained by linear regression. log10 *K*_ow_ of predicted substance is compared to the range of log *K*_ow_ values in each structural class of the training set, and it internally defines the ADs. The lowest predicted values were used to validation.

#### T.E.S.T

T.E.S.T. estimates acute aquatic toxicity using several QSAR methodologies: hierarchical clustering, single model, the Food and Drug Administration method, multilinear regression method, group contribution method, mode of action method, nearest neighbour method and consensus methods. In the default consensus methods (used to validation), the predicted toxicity is simply the average of the predicted toxicities from the above QSAR methodologies (taking into account the applicability domain of each method). T.E.S.T. is trained on the endpoint from the EPA ECOTOX database [39]. T.E.S.T has AD for each method and a final AD where predicitons must be made by at least 2 methods for a consensus value to be used. If only a single QSAR methodology can make a prediction, the predicted value is deemed unreliable and not used. So if there is a predicted value given by consensus methods, we defined this situation as in the AD.

#### VEGA

VEGA provides seven models to predict the fish acute toxicity: (1) SarPy/IRFMN (V1.0.2), QSAR classification model based on fragments built by SarPy software. (2) KNN/Read-Across (V 1.0.0), Read-Across model. (3)NIC (V1.0.0), QSAR quantitative modely based on a Neural Network. (4) IRFMN (V1.0.0), Quantitative model. (5) IRFMN/Combase (V1.0.0), Quantitative model, specific for biocides, developed by IRFMN for the Combase EU project. (6) EPA (V 1.0.7), QSAR model for Fathead Minnow LC50 (96 h), based on multiple linear regression. The model extends the original model implemented in the T.E.S.T. software. (7) KNN/IRFMN(V1.1.0). KNN model on fathead minnow.

VEGA provides two models to predict the daphnia acute toxicity: (1) EPA (1.0.7), QSAR model, based on multiple linear regression. The model extends the original model implemented in the T.E.S.T. software. (2) DEMETRA (1.0.4), Hybrid Model upon two ANNs and a single PLS for pesticides.

Two sets of fragments have been considered and implemented in VEGA and freely available: Functional Groups that account for 154 chemical groups, and Atom-Cantered Fragments (ACF), for 115 fragments, each one corresponding to a type of atom with different connectivity. The software to analyse the chemical space checks for the presence of the above mentioned Functional Groups and ACF, then reports, for each of these chemical features, the total number of matches, the number of matches in each class, and its percentage. The overall reliability of the prediction is measured by combining statistical values, elements of case based reasoning, and possibly presence of active substructures. The possible reasons of concern are underlined. All those considerations are weighted and summed up in an index (in 0–1) that is called Applicability Domain Index (ADI) [26].

**Fig. 1:**
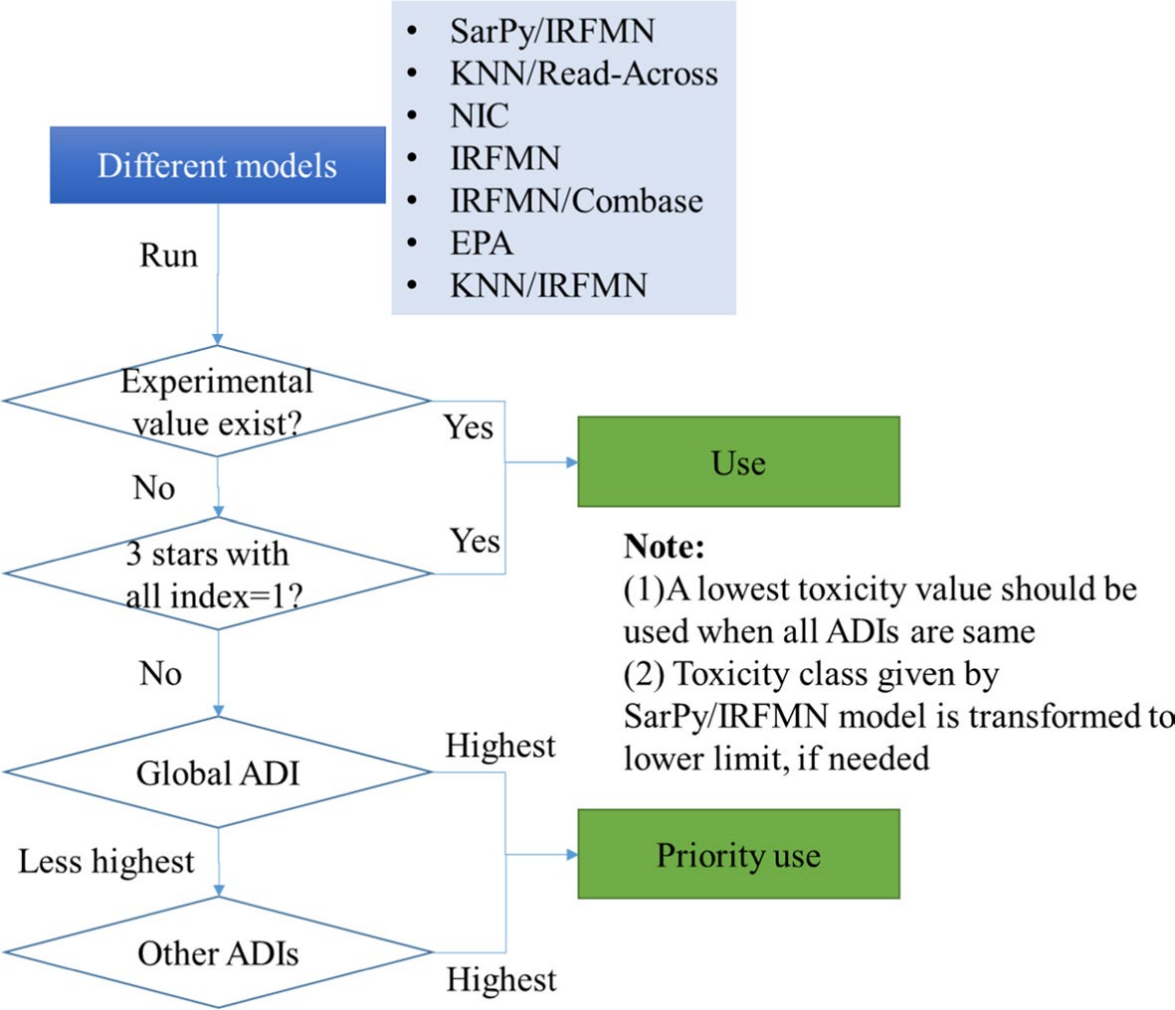
Recommended integrated assessment strategy with different models in VEGA when predicting the fish acute toxicity

All of the seven models predicting the fish acute toxicity and two models predicting daphnia acute toxicity were used with an integrated method (Fig. 1), except that experimental values were not used. The predicted results with good reliability were deem as inside the AD, else deem as outside the AD.

#### Danish Q. D

Danish Q. D. includes nearly all organic single constituent substances that were pre-registered or registered under REACH (around 80,000). The database was developed by Technical University of Denmark. The endpoints are modelled in two software systems (Leadscope, and SciQSAR), and an overall battery prediction is made to reduce “noise” from the individual model estimates and thereby improve accuracy and broaden the AD [27, 40].

Leadscope is a software program for systematic sub-structural analysis of a chemical using predefined structural features stored in a template library, training set-dependent generated structural features (scaffolds) and calculated molecular descriptors. Leadscope has a default automatic descriptor selection procedure. This procedure selects the top 30% of the descriptors (structural features and molecular descriptors) according to X2-test for a binary variable or the top and bottom 15% descriptors according to t-test for a continuous variable. After selection of descriptors the program performs partial least squares (PLS) regression for a continuous response variable, or partial logistic regression for a binary response variable, to build a predictive model.

The SciQSAR software provides over 400 built-in molecular descriptors such as connectivity indices, electrotopological (atom E and HE-state) indices, and other descriptors. For continuous data, regression analysis is used to build the predictive model, and a number of different regression methods are available such as regression on principal components and PLS.

The Battery results were used firstly. If not given for Battery results, the lowest toxicity value of Leadscope and SciQSAR was selected to verification.

#### Trent Analysis and Read Across

OECD QSAR Toolbox finds structurally and mechanistically defined analogues and chemical categories, which serve as sources for Read Across, Trent Analysis and QSAR for filling in data gaps. QSAR Toolbox has multiple functions, such as identifying analogues of a chemical, retrieving the existing experimental results of those analogues, and filling in data gaps through Read Across, Trent Analysis or QSAR.

The predictions of Read Across and Trent Analysis were accomplished by collecting a set of test data for PCCs considered to be in the same category as the target molecule. The category was firstly defined using categorization method of “Organic functional groups (nested)”. The analogues of each PCCs were identified. Then all available experimental data on 48 h-LC_50_ value for daphnia and 96 h-LC_50_ value for *Actinopterygii* of identified analogues were retrieved from the selected databases (Aquatic ECETOC, Aquatic Janpan MoE, Aquatic OASIS, ECHA REACH, ECOTOX and Food TOX Hazard EFSA). Finally the Read Across and Trend Analysis were implemented with internal standardized workflow. By default of Read Across, the QSAR Toolbox averages the result of the 5 “nearest” analogues (log10 *K*_ow_ in this case) to estimate the result for the target chemical. AD of each prediction was recorded as it automatic assessed by combing the log10 *K*_ow_ range and organic functional groups similarity. log10 *K*_ow_ must be in the range of all collected analogues, and organic functional groups must be included by that of all collected analogues.

### Statistical analysis

Two types of method were used to quantify the performance of all the models to PCCs: qualitative assessment and quantitative assessment methods. Only qualitative assessment was used to quantify the performance of the five models to NCs, as most of NCs were not harmful and only a limit test result of 96-h LC_50_ > 100 mg/L were given.

Qualitative effect assessment only needs classified chemicals according to toxicity values (Table 2). This is related to the toxicity classes described in the The Globally Harmonized System of Classification and Labelling of Chemicals (GHS) [41]. These classification criteria are accepted by most of countries as regulatory classes. In qualitative assessment, the experimental data and predicted data were classified into four classes based GHS criteria of United Nations (Table 2). If the predicted value and the experimental value are in the same regulation category, the prediction can be considered accurate without specific values.

**Table 2:** Classification criteria of acute toxicity according to GHS

Toxicity range (mg/L)	Class
LC_50_ ≤ 1	1 (very toxic)
1 < LC_50_ ≤ 10	2 (toxic)
10 < LC_50_ ≤ 100	3 (harmful)
LC_50_ > 100	4 (not harmful)

Quantitative assessment needs exact toxicity value to obtain the risk quotient [42]. In quantitative assessment, the difference between predicted and measured LC_50_ value was analysed, with difference factors of 10, 100 and 100.

A number of summary statistics were calculated to compare model performance. The correlation coefficient (*R*^2^), correlation coefficient of the AD (*R*^2^_AD_), root mean square error (RMSE), and percent of accuracy between predicted and measured toxicity were statistic with Microsoft excel. Software of IBM SPSS Statistics (V19) was used to obtain distribution of difference frequency between log10 experimental LC_50_ and log10 estimated LC_50_.

Total accuracy was calculated as:

$$\mathrm{Total}\ \mathrm{accuracy}=\frac{\mathrm{No}.\mathrm{of}\ \mathrm{correct}}{\mathrm{No}.\mathrm{of}\ \mathrm{all}-\mathrm{No}.\mathrm{of}\ \mathrm{missing}\ \mathrm{predictions}}\times 100\%$$

Similar to total accuracy, predictive power measures the total number of correct category assignments. However, lack of prediction was treated as an incorrect assignment:

$$\mathrm{Predictive}\ \mathrm{power}=\frac{\mathrm{No}.\mathrm{of}\ \mathrm{correct}}{\mathrm{No}.\mathrm{of}\ \mathrm{all}}\times 100\%$$

## Results

### Statistical distribution of experimental values

**Fig. 2:**
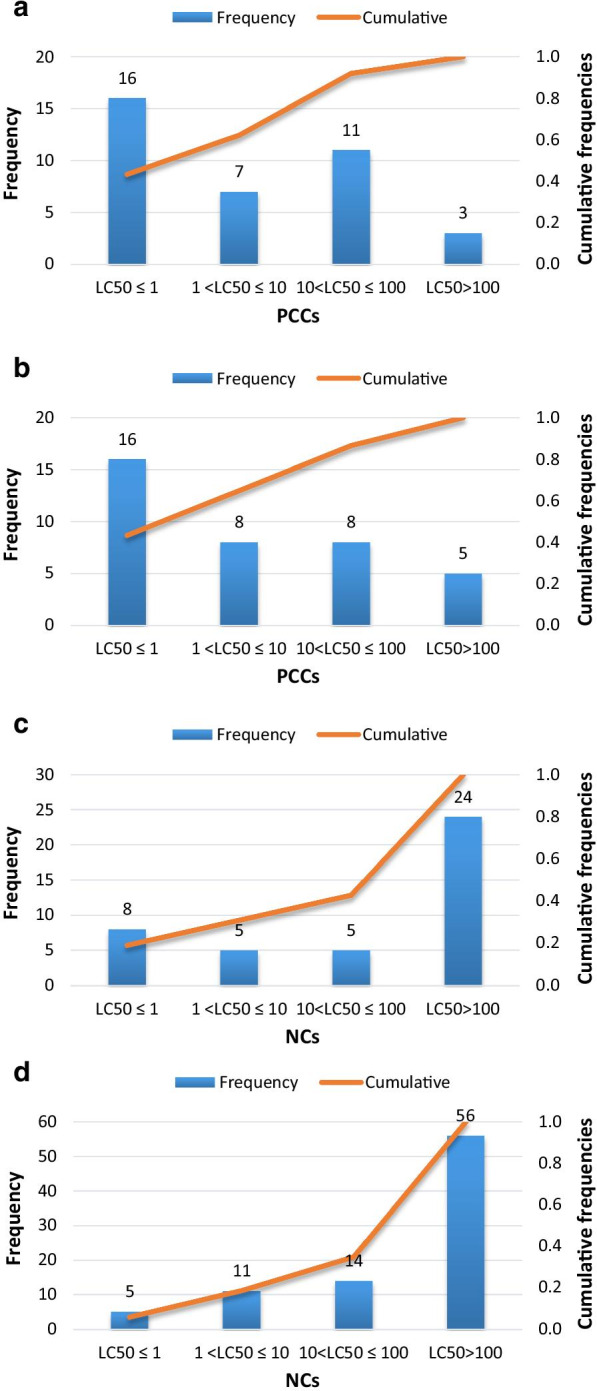
Distribution of acute toxicity of experimental values (mg/L). a 48-LC_50_ of daphnia for PCCs. b 96-h LC_50_ of fish for PCCs. c 48-LC_50_ of daphnia for NCs. b 96-h LC_50_ of fish for NCs.

The 37 PCCs assessed in this study represent a diverse array of commercial substances. They include olefins, nitrobenzene, perfluorinated and polyfluoro compounds, halogenated hydrocarbon, halogenated benzene, organophosphate, phenols, aldehydes, organophosphate, phthalates, polycyclic aromatic hydrocarbons. The experimental LC_50_ values of 37 chemicals cover all regulatory categories (Fig. 2 (A) and (B)). 43% of chemicals are very toxic chemicals. The number of very toxic, toxic and hazardous chemicals are account for 92 and 86% of all the chemicals for daphnia and fish acute toxicity, respectively.

The NCs assessed in this study include almost all of the organic functional groups. They are much more complex as many of which have two or more functional groups, and the most complex NC have 12 functional groups. The overall toxicity of NCs are lower than PCCs shown in Fig. 2 (c) and (d). The number of non-toxicity NCs account for 57 and 65% of total NCs to *Daphnia* and fish, respectively.

### Acute toxicity of daphnia

Experimental and predicted toxicity values to daphnia for the 37 PCCs are shown in Table 3, for the results of NCs can be found in section of “Availability of data and materials”.

**Table 3:** Experimental and predicted toxicity values to daphnia for the 37 PCCs

CAS No.	Chemical name	Exp.	ECOSAR		T.E.S.T.		Danish Q.D.		VEGA		Read Across		Trend Analysis		KATE	
		LC_50_	LC_50_	AD	LC_50_	AD	LC_50_	AD	LC_50_	AD	LC_50_	AD	LC_50_	AD	LC_50_	AD
120–82-1	1,2,4-Trichlorobenzene	1.68	1.88	In	2.88	In	1.04	In	2.69	In	5.36	In	171	In	1.60	In
81–15-2	Musk xylene	0.15	45.7	In	2.41	In	1.38	In	6.71	Out	6.90	In	0.55	InIn	NA	
75–09-2	Dichloromethane	27.0	146.0	In	59.06	In	NA		81.92	In	148	InIn	115	In	51.00	In
50–00-0	Formaldehyde	29.0	12.0	In	NA		NA		NA		2610	In	8090	In	NA	
77–47-4	Hexachlorocyclopentadiene	0.039	0.21	In	1.04	In	0.11	Out	0.07	Out	0.11	Out	NA		NA	
25,637–99-4	Hexabromocyclododecane	0.0032	0.0035	Out	0.16	In	0.01	Out	0.43	Out	0.17	Out	−84.0	Out	0.00	Out
91–20-3	Naphthalene	1.96	5.94	In	8.14	In	3.26	Out	0.24	In	11.5	In	99.0	AD	3.90	In
1763-23-1	Heptadecafluorooctanesulfonic acid	37.04	16.9	In	NA		18.20	Out	12.01	Out	63,800	In	29,900	In	NA	
307–35-7	Perfluoro-1-octanesulfonyl fluoride	100	0.0051	Out	NA		19.98	In	2.51	Out	NA		NA		NA	
2795-39-3	Potassium perufluorooctane sulfonate	27.0	16.9	In	NA		19.98	In	NA		12,000	In	17,500	In	NA	
25,154–52-3	Nonylphenol	0.14	0.168	In	0.55	In	0.32	In	3.88	Out	3.58	In	0.57	In	0.12	In
9016-45-9	NP-poly(ethyleneoxy)ethanol	0.15	0.211	Out	2.58	In	3.15	In	1.03	Out	12.70	Out	21.80	Out	0.09	Out
67–66-3	Chloroform	29.0	143	In	77.40	In	NA		17.07	Out	198	In	110	In	63	Out
79–01-6	Trichloroethylene	43.0	7.91	In	36.10	In	11.08	Out	5.27	Out	8.51	In	12.80	In	10	In
127–18-4	Tetrachloroethylene	18.2	3.68	In	9.86	In	4.13	Out	21.92	In	20.5	In	21.7	In	4.6	In
75–07-0	Acetaldehyde	12,418	32.6		829	In	583	In	117	Out	126	Out	− 451	Out	NA	
732–26-3	2,4,6-Tri-tert-butylphenol	0.072	0.108	In	0.0936	In	0.806	In	19.23	Out	3.16	Out	−3.32	Out	0.16	In
68,937–41-7	Isopropylphenyl phosphate	1.5	0.0044	Out	0.0115	In	0.00074	Out	0.0002	Out	1.41	In	2.91	In	NA	
50–32-8	Benzo[def]chrysene	0.25	0.0016	Out	0.2	In	0.225	In	0.1512	Out	NA		NA		NA	
120–12-7	Anthracene	0.0356	0.809	In	0.7	In	1.21	In	0.1007	In	0.173	Out	0.055	NA	0.8	In
56–55-3	Benz[a]anthracene	0.14	0.101	Out	0.28	In	0.359	In	0.1239	Out	0.25	Out	NA		0.0074	Out
53–70-3	Dibenz[a,h]anthracene	0.000551	0.0044	Out	0.11	In	0.138	In	0.0768	Out	0.198	Out	NA		0.012	Out
106–46-7	1,4-Dichlorobenzene	2.2	5.45	In	4.16	In	3.07	In	5.9	In	6.83	In	303	In	3.8	In
608–93-5	Pentachlorobenzene	0.18	0.203	Out	0.65	In	0.641	In	0.7177	Out	108	In	53.9	In	0.27	In
71–43-2	Benzene	10	36.9	In	49.1	In	7.65	Out	19.34	Out	181	In	2.26E7	In	16	In
108–88-3	Toluene	3.78	14.8	In	25	In	5.44	In	15.79	Out	185	In	56.8	In	4.5	In
115–96-8	Tris(2-chloroethyl) phosphate	170	135	In	0.0403	In	0.0857	In	0.0004	Out	104	Out	113	Out	NA	
117–81-7	Bis(2-ethylhexyl) phthalate	0.37	0.01	Out	0.99	In	7.06	Out	0.0299	Out	0.0363	In	0.0696	In	0.027	Out
84–74-2	Dibutyl phthalate	0.5	1.75	In	6.61	In	17.5	In	1.742	In	6.38	In	73.6	In	3	In
85–68-7	Benzyl butyl phthalate	0.74	1.4	In	3.17	In	9.92	In	0.0767	Out	2.84	In	4.96	In	2.5	In
84–69-5	Diisobutyl phthalate	3	2.17	In	6.44	In	26.2	In	0.1047	Out	0.0204	Out	−0.0906	Out	3.7	In
78–87-5	1,2-dichloropropane	55.9	32.2	In	50.1	In	13.1	Out	150.2	In	15.8	Out	NA		14	In
75–35-4	1,1-Dichloroethylene	37	12	In	10.3	In	15.2	Out	19.49	In	75	In	59	In	15	In
121–14-2	2,4-Dinitrotoluene	34.9	31.8	In	4.06	In	8.04	In	7.82	Out	7.92	In	30.8	In	0.27	In
95–53-4	o-Toluidine	0.52	23.3	In	1.49	In	6.82	In	6.48	Out	9.13	Out	11.8	Out	NA	
335–67-1	Pentadecafluorooctanoic acid	202	7.44	In	10.3	In	37.2	In	1.14	Out	1.01E6	Out	1.02E6	Out	NA	
87–86-5	Pentachlorophenol	0.73	0.711	In	0.19	In	0.769	Out	1.29	In	0.748	Out	0.886	Out	0.61	In

#### Models performance across the entire data set

Model performance was evaluated on the entire 37 PCCs and 42 NCCs. The performance metrics for all models tested in this evaluation to acute toxicity of daphnia are summarized in Table 4.

**Table 4:** Tool performance and comparison summary statistics to 48 h-LC_50_ of daphnia based on entire dataset

Chemicals	Methods	Measures of predictive performance	ECOSAR	T.E.S.T.	Danish Q.D.	VEGA	Read Across	Trend Analysis	KATE
37 PCCs	Number of missing predictions		0	4	3	2	2	6	12
	Qualitative	Number of correct	24	21	20	18	16	14	21
		Number of incorrect	13	12	14	17	19	17	4
		Total accuracy (%)^a^	65	64	59	51	46	45	84
		Predictive power (%)^b^	65	57	54	49	43	38	57
		*R*^2^ (toxicity class)	0.46	0.46	0.37	0.29	0.51	0.33	0.65
	Quantitative	Accuracy within a factor of 10 (%)	76	67	68	63	49	45	80
		Accuracy within a factor of 100 (%)	86	91	91	80	83	55	96
		Accuracy within a factor of 1000 (%)	97	97	94	94	94	81	100
		*R*^2^(log10 LC_50_)	0.40	0.42	0.38	0.13	0.42	0.40	0.68
42 NCs	Qualitative	Number of correct	22	9	13	9	/^c^	/	9
		Number of incorrect	16	22	11	32	/	/	11
		Number of missing predictions	4	11	18	1	/	/	22
		Total accuracy (%)^a^	58	29	54	22	/	/	45
		Predictive power (%)^b^	52	21	31	21	/	/	21
		*R*^2^ (toxicity class)	0.35	0.04	0.50	0.04	/	/	0.36

^a^ Total accuracy is the fraction of chemicals assessed by each tool for which the predicted LC_50_ falls within the same regulatory category as the measured LC_50_. ^b^ Similar to total accuracy, predictive power measures the total number of correct category assignments. However, lack of prediction is treated as an incorrect assignment. ^c^ Not analyzed

##### Prediction to 37 PCCs

In qualitative assessment based on classification into the four toxicity classes of the entire 37 PCCs data set, KATE has total accuracies of 84%, which is highest among all of the test models. However, the predictive power of KATE is decrease to 57% as it did not predict 12 of PCCs, which is most among all of the test models. ECOSAR predict all of the PCCs, both of total accuracy and the predictive power is 65%. Based on total accuracies, the tested tools can be ranked in the following order from highest- to lowest-performers: KATE > ECOSAR >T.E.S.T. > Danish Q.D. > VEGA>Read Across>Trend Analysis. KATE shows the excellent performance as only five PCCs were predicted incorrectly.

In quantitative assessment based on comparison of the LC_50_ value of PCCs provided by models, the KATE and ECOSAR shows better performance with accuracies of 80 and 76%, respectively, when predictions fall within a factor 10 of the measured LC_50_. All of the models can achieve the accuracy of 80% when differences between measured and predicted toxicity within a factor 100, except for Trent Analysis was only 55%. From Coefficient of variance (*R*^2^) in both qualitative assessment and quantitative assessment, it can be further prove that KATE has the best performance.

##### Prediction to 42 NCs

In qualitative assessment based on classification into the four toxicity classes of the entire 42 NCs dataset, total accuracy and predictive power are decrease dramatically compare with to PCCs. Danish Q.D and KATE have 18 and 22 chemicals that could not be predicted, which are relative higher than other model. These indicate that the performance of models are poor to NCs, and predictive power to NCs is limited.

#### Model performance within AD

Robust and relevant AD definition is essential for model performance. Model performance within ADs is shown in Table 5.

**Table 5:** Model performance to 48 h-LC_50_ of daphnia for chemicals within each applicability domains

Chemicals	Method	Measures of predictive accuracy	ECOSAR	T.E.S.T.	Danish Q. D.	VEGA	Read Across	Trend Analysis	KATE
37 PCCS	General	Number of inside AD	27	22	22	10	21	22	19
		Number of outside AD and missing prediction	10	4	15	27	16	15	18
	Qualitative	Number of correct	17	21	12	6	6	9	15
		Number of incorrect	10	12	10	4	15	13	4
		Accuracy inside AD (%)	63	64	55	60	29	41	79
		Coefficient of variance (*R*^2^_AD_)	0.40	0.46	0.28	0.58	0.29	0.54	0.53
	Quantitative	Accuracy within a factor of 10 (%)	85	67	59	100	52	55	89
		Accuracy within a factor of 100 (%)	96	91	91	100	86	64	95
		Accuracy within a factor of 1000 (%)	100	97	96	100	95	96	100
		RMSE (log10 scale)	0.82	0.91	1.24	0.48	1.49	2.06	0.70
		*R*^2^_AD_ (log10 LC_50_)	0.51	0.42	0.43	0.82	0.35	0.36	0.51
57 NCs	Qualitative	Number of inside AD	32	31	13	10	/^a^	/	10
		Number of outside AD and missing prediction	10	11	29	31	/	/	32
		Number of correct	20	9	7	3	/	/	4
		Number of incorrect	12	22	6	7	/	/	6
		Accuracy inside AD (%)	63	29	54	30	/	/	40
		Coefficient of variance (*R*^2^_AD_)	0.45	0.04	0.76	0.09	/	/	0.66

^a^ Not analyzed

##### Prediction to 37 PCCs

ECOSAR has the most chemicals inside the AD, with 27 of the 37 PCCs. VEGA has the least chemicals inside the AD, with 10 of the 37 tested chemicals, showing a rigorous AD assessment mechanism.

In qualitative assessment, the accuracies of VEGA increased slightly from 51 to 60% after considering AD. T.E.S.T. kept at 64%. The accuracies of other five tools did not increase when inside the AD.

Accuracies and R^2^_AD_ of Danish Q.D., Read Across and KATE after considering the AD are decreasing. Some PCCs with correct predicted were excluded as a results of outside the AD. Danish Q.D., Read Across and KATE assess the AD by the range of log10 *K*_ow_ and structural classes, and the methods are not as rigorous as used by VEGA. Similar phenomena was also found by Melnikov et.al [43]. that KATE total accuracy decreased from 58 to 46% when analysis is limited to the compounds within its AD.

In quantitative assessment, performance of all tools is increase when inside the AD. VEGA shows the best performance with 100% accuracy when predictions fall within a factor 10 of the measured LC_50_. VEGA also has the lowest RMSE (0.48 log10 units) and highest R^2^_AD_ (0.82). Read Across and Trent Analysis have the worst predictive ability from all of the indictors: accuracies, RMSE and R^2^_AD_.

In general, Based on the accuracies of quantitative assessment, the tested tools for daphnia can be ranked in the following order, from the highest to the lowest performers: VEGA> KATE > ECOSAR > T.E.S.T. > Danish Q.D > Trend Analysis > Read Across.

##### Prediction to 57 NCs

The number of NCs outside the AD and missing prediction are more for Danish Q.D, VEGA and KATE, except for ECOSAR and T.E.S.T. Accuracies inside AD of ECOSAR and Danish Q. D. are still high as same as in prediction to PCCs, whereas, T.E.S.T., VEGA and KATE are lower with accuracies of 29, 30 and 40%, respectively.

**Fig. 3:**
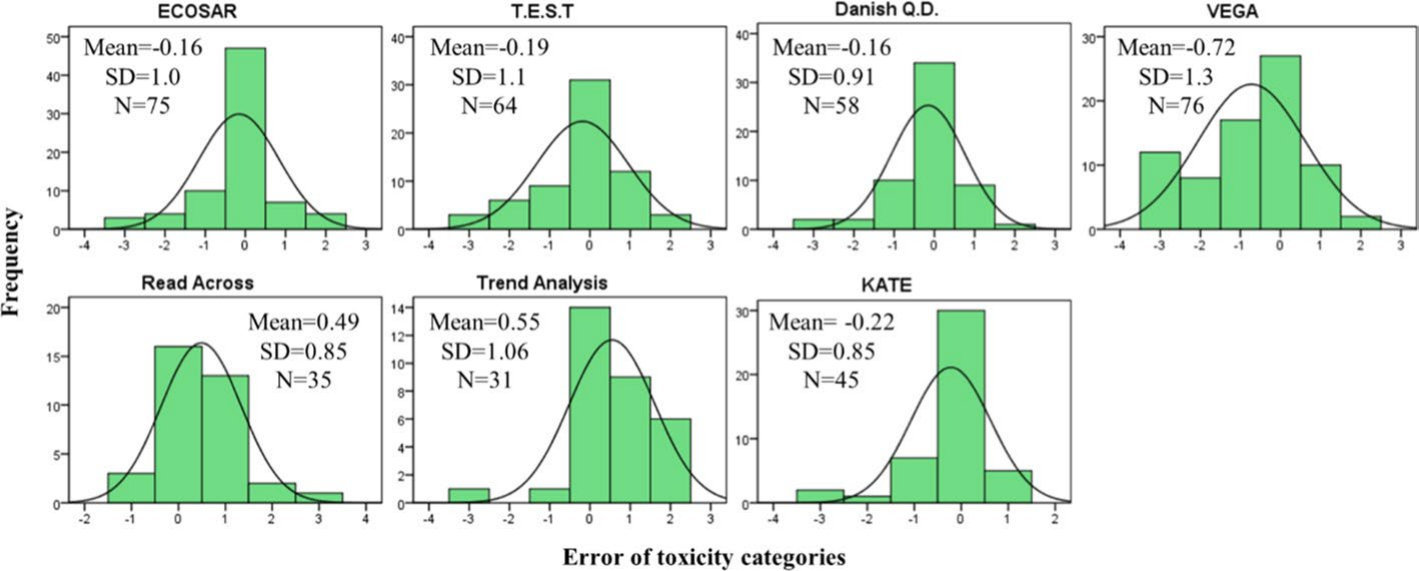
Errors distribution (predicted – experimental) of daphnia toxicity categories. Positive errors indicate predicted LC_50_ is above experimental LC_50_ and toxicity was underestimated. Dataset of Read Across and Trend Analysis were based on PCCs, others were based on both PCCs and NCs. Mean is average error, SD is Standard Deviation, and N is number of chemicals.

Figure 3 shows the error distribution of the daphnia toxicity predictions to PCCs and NCs with respect to under- and overestimation. Positive errors indicate predicted LC_50_ is above experimental LC_50_ and toxicity is underestimated. Considering the error of prediction between the log10 LC_50_ of the experimental value and the log10 LC_50_ of the estimated toxicity value provided by the model, over- and underestimation of daphnia by ECOSAR, T.E.S.T, Danish Q.D. and KATE are more or less similarly distributed. Daphnia toxicity predicted by VEGA appear to be overestimated, whereas, Read Across and Trent Analysis are underestimated significantly. Underestimated toxicity does not meet the principal of reasonable worst-case.

### Acute toxicity of fish

Experimental and predicted toxicity results to fish for the 37 PCCs are shown in Table 6, for the results of 86 NCs can be found in section of “Availability of data and materials”.

**Table 6:** Experimental and predicted toxicity results to fish for the 37 PCCs

CAS No.	Chemical name	Exp.	ECOSAR		T.E.S.T.		Danish Q.D.		VEGA		Read Across		Trend Analysis		KATE	
		LC_50_	LC_50_	AD	LC_50_	AD	LC_50_	AD	LC_50_	AD	LC_50_	AD	LC_50_	AD	LC_50_	AD
120–82-1	1,2,4-Trichlorobenzene	0.7	2.77	In	1.84	In	1.45	In	2.67	In	15.3	In	244	In	2.4	In
81–15-2	Musk xylene	0.2	6.12	In	0.07	In	1.03	In	10.1	In	1.5	In	32	In	NA	
75–09-2	Dichloromethane	330	273	In	317	In	NA		251	In	2770	In	329		85	In
50–00-0	Formaldehyde	23.9	11.2	In	NA		NA		122	In	26	In	141	In	NA	
77–47-4	Hexachlorocyclopentadiene	0.007	0.217	In	0.33	In	0.25	Out	0.0227	In	0.164	Out	NA		NA	
25,637–99-4	Hexabromocyclododecane	0.0025	0.0037	Out	0.045	In	0.0013	Out	0.0135	Out	575	Out	411	In	0.0039	Out
91–20-3	Naphthalene	0.96	9.39	In	7.27	In	5.07	In	3.74	In	48	In	901	In	6.8	In
1763-23-1	Heptadecafluorooctanesulfonic acid	68	23.7	In	0.57	In	177,032	In	1.79	Out	47.1	In	237	In	2.6	In
307–35-7	Perfluoro-1-octanesulfonyl fluoride	4.7	0.045	Out	0.24	In	177,032	In	0.5612	Out	NA		NA		NA	
2795-39-3	Potassium perufluorooctane sulfonate	9.5	23.7	In	NA		177,032	In	NA		111	In	299	In	NA	
25,154–52-3	Nonylphenol	0.128	0.068	In	0.63	In	0.11	Out	0.5702	In	20.4	In	51.3	In	0.11	In
9016-45-9	Nonylphenoxypoly(ethyleneoxy)ethanol	5	0.274	Out	0.72	In	0.417	In	0.592	Out	22.4	In	4.1	In	0.35	Out
67–66-3	Chloroform	121	2464	In	72.24	In	NA		100.1	In	672	In	319	In	53	In
79–01-6	Trichloroethylene	44.52	9.48	In	30.49	In	9.95	Out	31.25	In	18.3	In	20.3	In	20	In
1163-19-5	Decabromodiphenyl oxide	0.183	6.6E-7	Out	6.4E-4	In	0.0004	Out	0.7998	Out	0.689	Out	NA		2.0E-6	Out
127–18-4	Tetrachloroethylene	8.4	4.27	In	15.65	In	2.86	Out	11.3	In	18.3	In	20.3	In	11	In
75–07-0	Acetaldehyde	30.8	29	In	36.99	In	134	Out	126.79	In	41.1	In	169	In	NA	
732–26-3	2,4,6-tri-tert-butylphenol	0.048	0.034	In	0.21	In	0.053	Out	0.8443	In	40.9	In	233	In	NA	
68,937–41-7	Isopropylphenyl phosphate	10.8	0.0045	Out	0.0184	In	0.0017	Out	3.75	no	84.2	In	89.8	In	0.0011	Out
120–12-7	Anthracene	2.78	1.15	In	0.6	In	1.2	In	1.42	In	4480	In	6880	In	1.6	In
106–46-7	1,4-Dichlorobenzene	1.24	8.52	In	4.19	In	5.51	In	5.41	In	23.3	In	248	In	5.8	In
608–93-5	Pentachlorobenzene	0.31	0.266	Out	0.41	In	0.12	In	0.316	In	22	In	76.7	In	0.38	In
118–74-1	Hexachlorobenzene	0.119	0.0068	Out	0.14	In	0.027	In	0.5434	In	41.8	In	110	In	0.2	In
71–43-2	Benzene	5.3	65.1	In	39.35	In	26.7	Out	30.39	In	77.2	In	270	In	40	In
108–88-3	Toluene	5.5	24.8	In	35.5	In	19.8	In	8.37	In	104	In	152	In	20	In
115–96-8	Tris(2-chloroethyl) phosphate	66	62.5	In	14.5	In	5.57	Out	2.38	Out	64.3	In	124	In	NA	
117–81-7	Bis(2-ethylhexyl) phthalate	0.16	0.0097	Out	0.33	In	0.0027	Out	54.09	In	204	In	173	In	0.011	Out
84–74-2	Dibutyl phthalate	0.48	1.11	In	1.11	In	0.339	In	1.13	In	0.408	In	60.9	In	1.3	In
85–68-7	Benzyl butyl phthalate	0.51	0.911	In	0.47	In	0.123	In	0.5976	In	110	In	116	In	1.1	Out
84–69-5	Diisobutyl phthalate	0.9	1.36	In	4.16	In	0.568	In	1.12	In	3.81	In	−28.9	In	1.6	In
78–87-5	1,2-Dichloropropane	133	55.4	In	61.8	In	42.3	Out	45.18	In	71.3	In	284	In	60	In
75–35-4	1,1-Dichloroethylene	107.9	14.8	In	64.1	In	49.1	Out	36.59	In	90.2	In	101	In	67	In
121–14-2	2,4-Dinitrotoluene	31	4.2	In	5.59	In	15.1	In	11.07	In	11	In	50.9	In	0.38	In
95–53-4	o-Toluidine	81.3	75.2	In	64.7	In	42.7	In	47.81	In	25.6	In	144	In	82	In
335–67-1	Pentadecafluorooctanoic acid	157	10.1	In	3.62	In	133,766	In	10.1	Out	1070	Out	1040	Out	1.2	In
87–68-3	Hexachlorobuta-1,3-diene	0.0949	0.171	In	0.56	In	2.6	Out	0.0252	In	NA		NA		NA	
87–86-5	Pentachlorophenol	0.25	0.477	In	0.37	In	0.174	In	0.3362	In	20.3	In	10.8	In	0.73	In

#### Model performance across the entire test set

Models performance were first evaluated on the entire dataset regardless of the AD to assess the tool utility for any new or existing chemical. The performance metrics for all models tested in this evaluation to acute toxicity of fish are summarized in Table 7.

**Table 7:** Tool performance and comparison summary statistics to 96 h-LC_50_ of fish based on entire dataset

Chemicals	Methods	Measures of predictive accuracy	ECOSAR	T.E.S.T	Danish Q.D.	VEGA	Read Across	Trend Analysis	KATE
37 PCCs	Number of missing predictions		0	2	3	1	2	4	9
	Qualitative	Number of correct	20	17	17	17	14	10	10
		Number of incorrect	17	18	17	19	21	23	18
		Total accuracy (%)^a^	54	49	50	47	40	30	36
		Predictive power (%)^b^	54	46	46	46	38	27	27
		*R*^2^ (toxicity class)	0.50	0.39	0.38	0.43	0.17	0.10	0.25
	Quantitative	Accuracy within a factor of 10 (%)	68	80	65	81	57	36	71
		Accuracy within a factor of 100 (%)	89	89	79	94	83	48	86
		Accuracy within a factor of 1000 (%)	92	97	85	97	94	76	89
		*R*^2^(log10 LC_50_)	0.31	0.35	0.27	0.34	0.32	0.03	0.21
86 NCs	Qualitative	Number of correct	34	25	17	18	/	/	17
		Number of incorrect	47	36	24	65	/	/	40
		Number of missing predictions	5	25	45	3	/	/	29
		Total accuracy (%)^a^	42	41	41	22	/	/	30
		Predictive power (%)^b^	40	29	20	21	/	/	20
		*R*^2^ (toxicity class)	0.08	0.10	0.13	0.001	/	/	0.03

^a^ Total accuracy is the fraction of chemicals assessed by each tool for which the predicted LC_50_ falls within the same regulatory category as the measured LC_50_. ^b^ Similar to total accuracy, predictive power measures the total number of correct category assignments. However, lack of prediction was treated as an incorrect assignment

##### Prediction to 37 PCCs

In qualitative assessment based on predictive power of classification into the four toxicity categories of the entire dataset, all models besides ECOSAR are performance not well, with accuracies not more than 50%. ECOSAR has the highest predictive power, with accuracy of 54% and all of the 37 chemicals predicted. The performance of ECOSAR to fish is similar as well as to daphnia. The total accuracies followed are Danish Q.D., T.E.S.T. and VEGA, with the accuracy of 50, 49 and 47%, respectively. Read Across and Trend Analysis have the lowest total accuracies, which are same as the situation of prediction to daphnia*.* The total accuracy of KATE is only 36%, the performance to predict the toxicity of fish is far less than prediction to daphnia.

In quantitative assessment of comparison log10 LC_50_ of experiment value with predicted value, VEGA and T.E.S.T. shows excellent predicted ability as they can achieve the accuracy of 80% when the absolute deviation between predicted and experimental value is limited to 10 times. The performance is followed by KATE and ECOSAR when deviation is limited to 10 times, with the accuracy of 71 and 68%, respectively. The coefficient of variance also reflect the same tendency with accuracy.

##### Prediction to 86 NCs

In qualitative assessment based on classification into the four toxicity classes of the entire 86 NCs, total accuracies decreased comparing with prediction to PCCs. As T.E.S.T., Danish Q.D and KATE could not predict 25, 45 and 49 NCs, respectively, the predictive power of these three tools are lowest. Both total accuracy and predictive power of VEGA are about 20%, which are decrease dramatically compare with prediction to PCCs. ECOSAR has the highest total accuracy and Predictive power compare with others tools, however, it is still not high with accuracy of about 40%.

#### Model performance within the AD

Model performance within AD to fish toxicity is shown in Table 8.

**Table 8:** Tool performance to 96 h-LC_50_ of fish for chemicals within each applicability domains

Chemicals	Methods	Measures of predictive accuracy	ECOSAR	T.E.S.T.	Danish Q. D.	VEGA	Read Across	Trend Analysis	KATE
37 PCCs	General	Number of inside AD	29	22	19	29	31	30	22
		Number of outside AD and missing prediction	8	15	18	8	6	6	15
	Qualitative	Number of correct	16	9	11	16	11	8	8
		Number of incorrect	13	13	8	13	20	23	14
		Accuracy inside AD (%)	55	41	58	55	35	26	36
		*R*^2^_AD_ (toxicity class)	0.66	0.41	0.58	0.57	0.07	0.06	0.35
	Quantitative	Accuracy within a factor of 10 (%)	83	82	74	90	55	42	86
		Accuracy within a factor of 100 (%)	100	95	79	97	81	55	95
		Accuracy within a factor of 1000 (%)	100	100	84	100	94	84	100
		RMSE (log10 LC_50_)	0.71	0.87	1.83	0.75	1.47	2.09	0.80
		*R*^2^_AD_ (log10 LC_50_)	0.68	0.52	0.57	0.68	0.14	0.00	0.50
86 NCs	Qualitative	Number of inside AD	58	61	22	50	/	/	21
		Number of outside AD and missing prediction	28	25	64	36	/	/	67
		Number of correct	32	25	13	18	/	/	7
		Number of incorrect	26	36	9	32	/	/	12
		Accuracy inside AD (%)	55	41	59	36	/	/	37
		*R*^2^_AD_ (toxicity class)	0.37	0.10	0.42	0.03	/	/	0.37

##### Prediction to 37 PCCs

The number PCCs inside the AD of VEGA, Read Across and Trend Analysis is most, with 29, 31 and 30 tested chemicals, respectively. T.E.S.T. and KATE have the minimal number of chemical inside the AD.

In qualitative assessment based on classification into the four toxicity categories, ECOSAR, Danish Q.D. and VEGA have the highest performance, with *R*^2^_AD_ of 0.66, 0.58 and 0.57 and accuracies of 55, 58 and 55%, respectively. The performance of tested tools for fish can be ranked in the following order, from the highest to the lowest performers: ECOSAR = Danish Q.D. = VEGA> T.E.S.T. > KATE > Read Across > Trend Analysis. The prediction Accuracies inside the AD is not significant improved in comparison to entire accuracy not considering the AD. This phenomenon is similar in prediction of daphnia.

In quantitative assessment, there are four models: VEGA, KATE, ECOSAR and T.E.S.T., with which the prediction accuracies are greater than 80% when the absolute error is limited to 10 times. VEGA reaches highest accuracy of 90%, with accuracy increased significantly after considering the AD. RMSE is a measure of accuracy, the lower of the RMSE, the higher of the predication accuracy. ECOSAR has the best RMSE (0.71 log10 units) and Trend Analysis has the worst (2.09 log units). All RMSEs of ECOSAR, T.E.S.T., VEGA and KATE are below 1 log10 scale, which are at same performance levels.

In general, based on the predictive power of quantitative assessment, the tested tools for fish can be ranked in the following order, from the highest to the lowest performers: VEGA > ECOSAR = KATE = T.E.S.T. > Danish Q.D > Read Across >Trend Analysis.

##### Prediction to 86 NCs

Accuracies inside AD of ECOSAR, T.E.S.T., Danish Q. D. and KAT are as same as prediction to PCCs. Whereas, Accuracy inside AD of VEGA to decreased from 55% for PCCs to 36% for NCs. The lower accuracy of VEGA’s prediction of NCs, probably because most of the measured results of SCs were non-toxic (LC_50_ > 100 mg/L), but when VEGA predicted, the lowest value of the 7 model included in VEGA was used and finally the probability of being predicted to be toxic category increased.

**Fig. 4:**
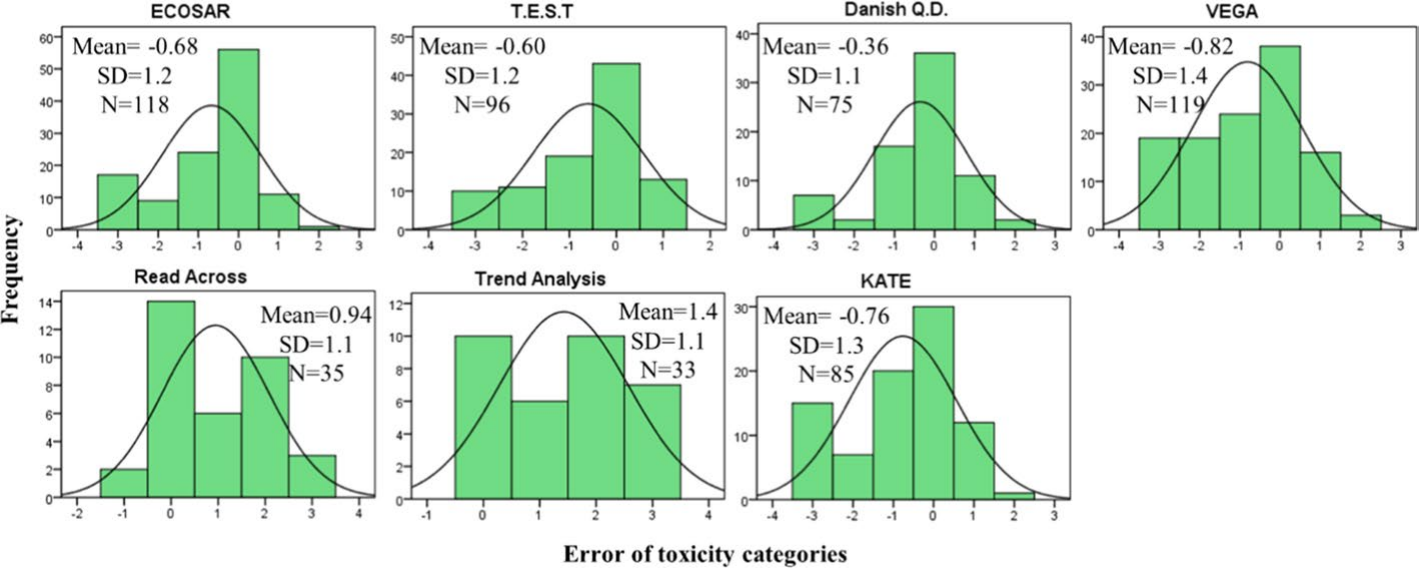
Errors distribution (predicted – experimental) of fish toxicity categories. Positive errors indicate predicted LC_50_ is above experimental LC_50_ and toxicity was underestimated. Datasets of Read Across and Trend Analysis were based on PCCs, others were based on both PCCs and NCs. Mean is average error, SD is Standard Deviation, and N is number of chemicals.

Figure 4 shows the distribution of the 96 h-LC_50_ fish toxicity predictions with respect to under- and overestimation. Positive errors indicate predicted LC_50_ is above experimental LC_50_ and toxicity is underestimated. Considering the error of prediction between the log10 LC_50_ of the experimental value and the log10 LC_50_ of the estimated toxicity value provided by the model, over- and underestimation of fish toxicities by Danish Q.D. are more or less similarly distributed. Fish toxicity predicted by ECOSAR, T.E.S.T, VEGA and KATE appear to be more often overestimated than underestimated, which meet the principal of reasonable worst-case.

## Discussion

### Methods to assess AD

All models provide AD assessments that predictions fall inside or outside the AD of the models. Most of these models (ECOSAR, KATE, Read Across and Trent Analysis) assess the AD directly with the range of log10 *K*_ow_. In addition to log10 *K*_ow_, these models also consider the structural similarity. The ECOSAR package provides warnings when the model prediction is above the substance solubility limit or if the substance log10 *K*_ow_ is outside the AD, it is helpful when non-professional application.

T.E.S.T. does not provide the AD of results directly. However, T.E.S.T has AD for each method and a final AD where predicitons must be made by at least 2 methods for a consensus value to be used .

Although there is no criterion to judge the validity or invalidity of the predicted data, predicted results within the AD are preferred. Although, the prediction accuracy inside the AD is not obviously improved compare to total accuracy not considering the AD in qualitative assessment, it improved significant in quantitative assessment.

There is no single and absolute AD assessment methods for a given model. Generally, the broader the definition of the AD, the lower the accuracies. This principle can be confirmed in the prediction of daphnia, in which the number of PCCs outside the AD and missing prediction are most by VEGA, however, the performance is best. In the quantitative evaluation within AD with the 10-fold factor, the accuracy of VEGA is the highest among all of the models, both to daphnia and to fish toxicity, with accuracy of 100 and 90%, respectively. The reason for the highest accuracy of VEGA prediction may be attributed to the detailed definition of the AD.

VEGA assess the AD with overall reliability, which is a relative complex mechanism. An overall reliability of the prediction is measured in a quantitative manner, whose value ranges from 1 to 0, by considering five factors, including Global AD Index, similar index of molecules with known experimental value, accuracy index of prediction for similar molecules, concordance index for similar molecules, index of Atom Centered Fragments similarity check. All those considerations are weighted and summed up into reliability of a model.

### Difference between classification and quantitative assessment

The qualitative method has a certain randomness for the substances at the classification boundary point. Substances at the toxicity boundary point will be divided into two distinct toxicities class easily. Therefore, qualitative method with toxicity classification method to assess accuracy will be inferior to quantitative methods in terms of scientific significance. The current aquatic acute classification method is based on the 10-fold factor in toxicity values. The quantitative method with a 10-fold factor is similar to the toxicity classification method, but it overcomes the uncertainty of the boundary points and is more meaningful for accuracy evaluation. It can also be proven from the results that the accuracy of the quantitative method is higher than that qualitative method. Therefore, the results of quantitative method is a good indicator to assess the performance of tested tools.

### Integrated assessment strategy when predicting the fish acute toxicity using VEGA

In the quantitative evaluation to prediction both daphnia and fish toxicity inside the AD, VEGA performs very well with the highest accuracy. However, there are seven models can be used to predict the fish acute toxicity in VEGA. Some confuse existing even if internal reliability is given. For example, several models may give the same liability with different AD index. And SarPy/IRFMN model is a classification model, it will give a toxicity class instead of toxicity value. Therefore, it is crucial to choose the most rational value of different models, and to use the toxicity class provided by SarPy/IRFMN model in quantitative effect assessment.

In order to make full advantage of VEGA, we proposed an integrated assessment strategy for fish acute toxicity, as shown in Fig. 1. This integrated assessment strategy were used in this study except that experimental values were not used, and it is prove to be useful.

- Step 1: if experimental value exist, it should be used, else go to step 2.
- Step 2: if reliability shows 3 stars with all ADI =1, it should be used, else go to step 3 at the following case:
  -If more than 1 models have 3 stars, or.
  -If models have only 2 stars or 1 star.
- Step 3: if it has a highest global ADI, it should be priority used, else go to step 4.
- Step 4: if the other ADI outperforms the others models, it should be priority used.

Notes: (1) A lowest toxicity value should be used when all ADIs are same; (2) Toxicity class given by SarPy/IRFMN model is transformed to lower limit, if needed. e.g. transformed the toxic-3 (between 10 and 100 mg·L^− 1^) to 10.1 mg·L^− 1^.

### QSAR vs Chemical category approach

ECOSAR, KATE, T.E.S.T. Danish Q.D and some of models in VEGA belong to QSAR methods. Both Read Across and Trent Analysis method are category approach. QSAR models and category approach method have similarities and differences.

In QSAR Toolbox, application strategy of Read across, Trend analysis and QSAR models is addressed. Read across is recommended for “qualitative” (e.g. skin sensitisation or mutagenicity) or “quantitative endpoints” (e.g., 96 h-LC_50_ for fish) if only a low number of analogues with experimental results are identified. Trend analysis is the appropriate data-gap filling method for “quantitative endpoints” (e.g., 96 h-LC_50_ for fish) if a high number of analogues with experimental results are identified. QSAR models can be used to fill a data gap if no adequate analogues are found for a target chemical.

The issue of chemical-to-chemical similarity is not directly present in the case of QSAR models. In the case of QSAR models, the target chemical is in some way compared with the whole population of chemicals as the basis of the model, and this is addressed within the AD of the model. Thus, the comparison is done not between one chemical and another, or a few others, as in the category approach, but with the whole set of compounds used for the model.

The overall structure of the SAR models model is like a collection of read across models, with similarity structure or fragment are collect and statistic. Identification of similarity structure in QSAR models is completed automatically. The evaluation of similar compound(s) in case of category approach is often done manually, typically done by the expert, which is quite subjective.

The accuracies of Read Across and Trend Analysis method are lowest among of tested tools. Read Across may be used when there are experimental data from high quality databases for one or more substances which are similar enough to the target chemical of interest. It is difficult to assess the quality of experimental data. Predictions applied in this research were based on category on organic functional groups, and standardized workflow in QSAR Toolbox. However, Trend Analysis can be further refined by subcategorization, such as elimination of analogues, which are dissimilar to the target chemical with respect to have same mode of action or same elements. Expert judgement always used when removing outliers. Each expert is guided by his or her past experience, pieces of information may escape her or his knowledge, the weight assigned to each element of evidence and value may be different, and expressed in a subjective way, such as likely, plausible, reasonable, level of concern, etc. and hence often difficult to replicate. Besides, the category approach is typically not so strictly formalized, depending on the similar chemicals data existing in internal database [44].

**Fig. 5:**
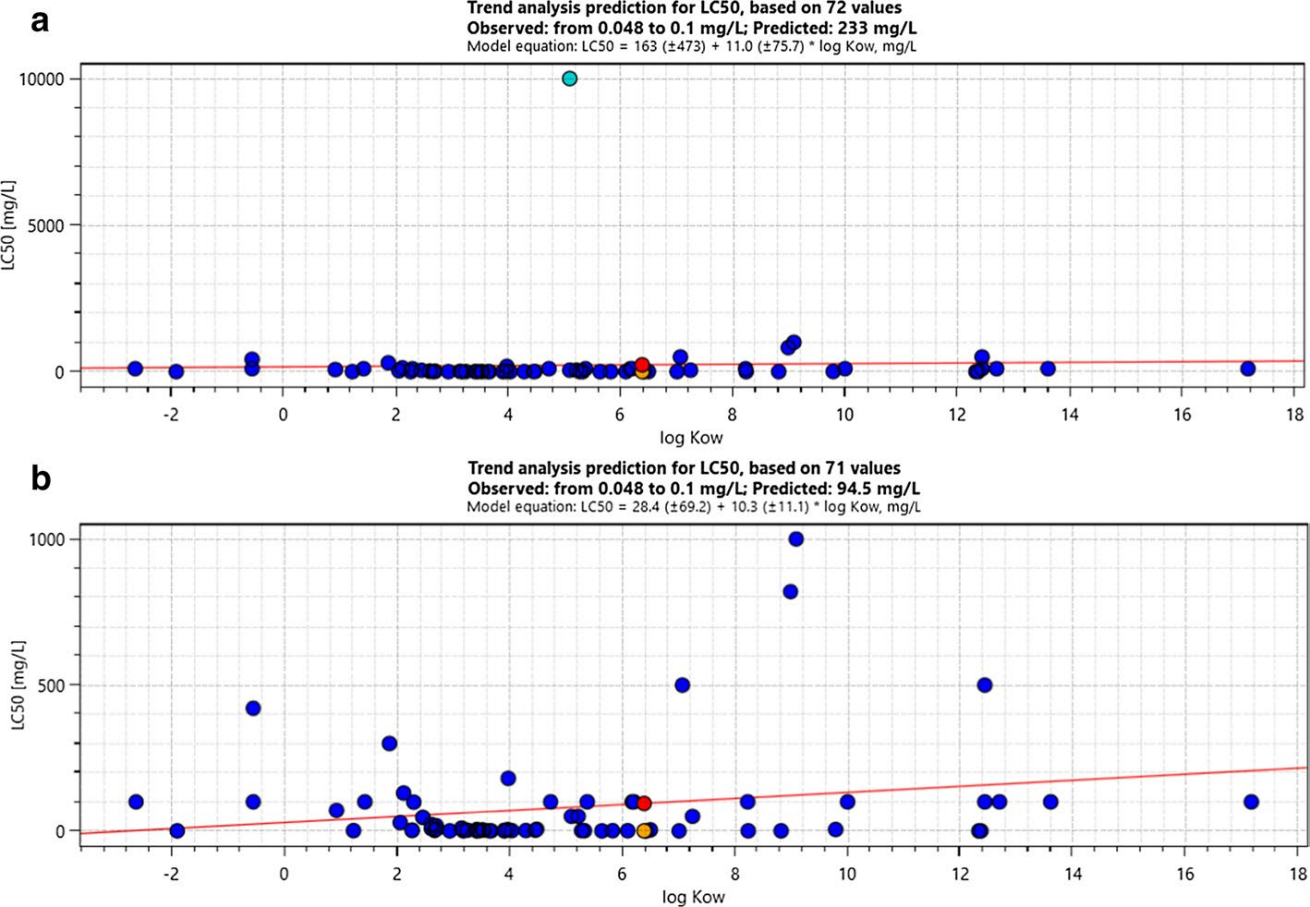
Case study on predicting fish 96-h LC_50_ to 2,4,6-tri-tert-butylphenol using Trent Analysis. (**a** Using standardized workflow in QSAR Toolbox without any manually disruption, **b** Using standardized workflow after deleting an obvious outlier substance)

A case study is shown in Fig. 5 that fish 96-h LC_50_ to 2,4,6-tri-tert-butylphenol was predicted using Trent Analysis. Figure 5a is the case that using standardized workflow in QSAR Toolbox without any manually disruption. An outlier can be judged easily. However, after deleting that obvious outlier, the result is still uncertain on how to refining shown in Fig. 5b. Thus, professional judgement require by chemical category methods limit application in regulation purpose, especially in high throughput screening in risk assessment. QSAR Toolbox also allows some different category methods, such as acute aquatic toxicity classification by ECOSAR, acute aquatic toxicity Mode of Action by OASIS, acute aquatic toxicity classification by Verhaar (Modified). Thus, performance of these category methods need further assessment, and they shall be used limiting in experts. At the same time, more intelligence technologies, such as artificial intelligence shall apply in category approach.

### PCCs that were incorrect predicted frequently

There are two PCCs, which daphnia toxicity were predicted incorrectly by more than 2 models (Table 9). The water solubility of anthracene is 0.047 mg·L^− 1^, which is lower than experimental LC_50_ value of 0.0356 mg·L^− 1^, indicating that experimental LC_50_ value may be tested incorrectly. There was only one experimental data of anthracene, so the acute toxicity to daphnia needs further testing.

**Table 9:** The PCCs that daphnia toxicity were predicted incorrectly by more than 2 models

Substance	Water solubility/mg·L^−1^	Experimental LC_50_/mg·L^− 1^		No. of models incorrect predicted
		Used to validation	Range in models and mean	
Anthracene	0.047	0.0356	0.0356	4
Dibutyl phthalate	11.2	0.5	1.4 ~ 3.7, 3.52	4

The experimental LC_50_ value to daphnia used to validate of dibutyl phthalate is 0.5 mg·L^− 1^, which was evaluated and accepted by ECHA. However, values are range from 1.4 to 3.7 mg·L^− 1^ gathered from database of these models. Predicted LC_50_ value of dibutyl phthalate from T.E.S.T, Danish Q.D, Read Across and Trend Analysis is 6.61, 17.5, 6.68 and 73.6 mg·L^− 1^, respectively. Therefore, it is the experiment value difference causing the “incorrectly prediction” to dibutyl phthalate by T.E.S.T, Danish Q.D and Read Across. Trend Analysis will still give a value that exceed to 10 times difference to experimental value, which performances not well.

For the acute toxicity of fish, according to the evaluation criterion that the difference between the experimental value and the predicted value is 10 times, there are 6 substances that more than 3 models predicted incorrectly, shown in Table 10.

**Table 10:** The PCCs that fish toxicity were predicted incorrectly by more than 2 models

Substance	Water solubility /mg·L^−1^	Experimental LC_50_/mg·L^− 1^		No. of models incorrect predicted
		Used to validation	Range in models and mean	
Musk xylene	0.15	0.2	2.9 ~ 47 (9.87)	3
Heptadecafluorooctanesulfonic acid	0.10	68	68	3
2,4,6-tri-tert-butylphenol	0.063	0.048	0.06 ~ 0.1 (0.07)	3
Benzene	1880	5.3	5.3 ~ 452 (83)	3
Bis(2-ethylhexyl) phthalate	0.27	0.16	0.16 ~ 1106 (573)	3
Pentadecafluorooctanoic acid	0.48	157	24.6 ~ 607 (316)	4

Among them, five substance have low water solubility of below 1 mg·L^− 1^. In principle, the experimental LC_50_ value of a substance should be lower than its water solubility. The water solubility of musk xylene, 2,4,6-tri-tert-butylphenol and bis(2-ethylhexyl) phthalate, show no significant difference to experimental LC_50_ value. Water solubility of heptadecafluorooctanesulfonic acid and pentadecafluorooctanoic acid is much lower than experimental LC_50_ value, indicating an incorrect experimental data. In fact, substance with low water solubility is classed as “difficult to test”, the aquatic toxicity of these difficult substance were often testing improperly even at GLP condition. Hence, the special caution should be given to this low water solubility substance when developing models. Meanwhile, uncertainly of models when validation and comparison of these PCCs, with low water solubility. As a result, some of the differences between model predictions and measured toxicity values can be partially attributed to the measured toxicity values themselves being less-than-perfect indicators of true toxicity. The errors associated with the measured toxicity values, however, should not affect our conclusions regarding the relative performance of the tested models (their rank orders), particularly in the common PCCs comparison, because all models are being evaluated against the same measured toxicity values.

Danish Q.D. predicted large errors to heptadecafluorooctanesulfonic acid, perfluoro-1-octanesulfonyl fluoride, potassium perufluorooctane sulfonate, pentadecafluorooctanoic acid, with which all LC_50_ value are above 100,000 mg·L^− 1^. There are two models in Danish Q.D: Leadscope and SciQSAR. As a case to predict Heptadecafluorooctanesulfonic acid, Leadscope predict a 0.00636 mg·L^− 1^, that is much closer to its water solubility of 0.10 mg·L^− 1^ than SciQSAR with predicted value of 354,065 mg·L^− 1^. This situation is similar in prediction of Perfluoro-1-octanesulfonyl fluoride, Potassium perufluorooctane sulfonate, Pentadecafluorooctanoic acid. Therefore, the SciQSAR model in Danish Q.D. is note suite for estimate the fish acute toxicity of perfluorinated compounds.

There are 54 experimental 96 h- LC_50_ fish values of benzene ranging from 5.3 mg·L^− 1^ to 542 mg·L^− 1^ collected in QSAR Toolbox, covering 21 fish species within the *Actinopterygii* class. As many factors affect the experimental results, such as test method, test conditions, species, or even the experience dealing with difficult substance.

It is difficulty to select a fish species to compare the models performance, as the fish species in tanning data of some model are not deterministic. Hence, this single point comparison method has some limitation when more than one experiment data exist. Therefore, we suggest that distribution of multiple data other than single value should be consider when developing in silico models.

### Analysis to Groups of NCs that were incorrect predicted frequently

The functional groups of NCs with more than three model prediction incorrectly were analyzed. Among them, the functional groups with more than 2 occurrences are shown in Table 11.

**Table 11:** Groups in NCs that were incorrect predicted frequently and the number of occurrences (≥2)

Daphnia toxicity		Fish toxicity	
Group name	n^a^	Group name	n^a^
Aryl	4	Aryl	9
Aryl halide	3	Aromatic amine	6
Aromatic amine	3	Organic amide and thioamide	6
Nitrile	2	Alkyl (hetero)arenes	6
Carbamate	2	Ketone	5
Alkyl (hetero)arenes	2	Diketone	4
Amidine	2	Aryl halide	4
Alcohol	2	Ether moiety	4
Organic amide and thioamide	2	Alkane,branched with secondary carbon	4
Alkyl-, alkenyl- and alkynyl (hetero)arenes	2	Amine,tertiary	3
Phenol	2	Alkene moiety	3
Alkane,branched with tertiary carbon	2	Alkyl halide	3
Pyrazolone	2	Alcohol	3
Carboxylic acid ester	2	Phenol	2
		Alkance,branched with quaternary carbon	2
		Alkane,branched with tertiary carbon	2
		Isopropyl	2
		Carboxylic acid ester	2
		Aliphatic amine,tertiary	2
		Azo	2
		Carboxylic acid	2

^a^n is the number of occurrences of a group that were incorrect predicted

Of the 42 NCs in the daphnia toxicology prediction, 14 substances were simultaneously incorrect predicted by more than 3 models. The most frequently predicted functional groups are aryl, aryl halide, and aromatic amine.

Of the 86 NCs in the fish toxicology prediction, 40 substances were simultaneously incorrect predicted by more than 3 models. The most frequently predicted functional groups are aryl, aromatic amine, organic amide and thioamide, alkyl (hetero)arenes, ketone, diketone, aryl halide, ether moiety, alkane branched with secondary carbon.

So these function groups should be pay more attention when developing in silico tools.

### Outlook

In silico tools are developed based on existing information to hazard. However, over 350,000 chemicals and mixtures of chemicals have been registered for production and use [1]. These chemicals consisted various type of chemicals. As science and technology advances, the chemicals synthetic or prepared chemicals are more and more complicated. Existing in silico tools have note covered all type of chemicals. It is expect that most of chemicals registered or used are not testing for their hazards, and hence no abundant data to support the development of in silico tools. Besides, in silico tools developed are most focus on individual compounds, it is difficulty to identified hazard of a number of mixtures, polymers and UVCBs, the number of which is over 75,000 [1].

So, testing is still needed whether it is used to identify chemical hazards or to provide more information to develop in silico tools. In silico tools are also need continuous development to accuracy, and expansion to AD of various substance, such as mixtures, polymers and UVCBs.

## Conclusion

In this study, the performance of seven in silico methods (ECOSAR, T.E.S.T., Danish Q. D., VEGA, KATE, Read Across and Trend Analysis) for acute aquatic toxicity to daphnia and fish was evaluated and compared using PCCs and NCs datasets.

In the quantitative evaluation of PCCs with the criteria of 10-fold difference between experimental value and estimated value, the accuracy of VEGA is the highest among all of the models, both in prediction of daphnia and fish acute toxicity, with accuracy of 100 and 90% after considering AD, respectively. The performance of KATE, ECOSAR and T.E.S.T. is at the similar level, with the accuracies are slight lower than VEGA. The accuracies of Danish Q.D. is lowest among above tools within them QSAR is the main mechanism. The performance of Read Across and Trent Analysis is lowest among all of the tested in silico tools by standardized workflow of QSAR Toolbox, indicating that chemical category approach shall limited in expert use at this stage. The main factor affects the accuracies of in silico tools may be the distribution of multiple experimental data, and the accuracies of experimental values for PCCs with poorly water solubility.

The performance of models to NCs that are much more complex are not as well as to PCCs, indicating in silico tools are also need continuous development. Testing is still needed whether it is used to identify hazards of NCs or to provide more information to develop in silico tools.

## Data Availability

The datasets used and/or analysed during the current study are available from the corresponding author or from: https://pan.baidu.com/s/19I6oMJDAhMDw2eatJ6EViA, with Extracted code: t392.

## Abbreviations

ACF: Atom-Cantered Fragments
AD: Applicability domain
ADI: Applicability Domain Index
ASTER: Assessment Tools for the Evaluation of Risk
Danish Q. D.: Danish QSAR Database
ECHA: European Chemical Agency
ECOSAR: Ecological Structure Activity Relationships
EPISuite: Estimation Programs Interface Suite
GHS: The Globally Harmonized System of Classification and Labelling of Chemicals
GLP: Good Laboratory Practice
KATE: Kashinhou Tool for Ecotoxicity
*K*_ow_: Octanol water partition coefficient
LC_50_: Median lethal concentration
MEP: Ministry of Environment Protection
NCs: New Chemicals
OASIS: Optimized Approach Based on Structural Indices Set
OECD: Organization for Economic Co-operation and Development
PBT/vPvB: Persistence, bioaccumulation, and toxicity/very persistent and very bioaccumulative
PCCs: Priority Controlled Chemicals in China
PLS: Partial least squares
QSAR: (Quantitative) Structure–Activity Relationships
*R*^2^: The correlation coefficient
*R*^2^_AD_: Correlation coefficient of the AD
REACH: Registration, Evaluation, Authorization and Restriction of Chemicals
RMSE: Root mean square error
SIMLES: Simplified Molecular Input Line Entry System
T.E.S.T.: Toxicity Estimation Software Tool
UVCBs: Chemical Substances of Unknown or Variable Composition, Complex Reaction Products and Biological Materials
VEGA: Virtual models for property Evaluation of chemicals within a Global Architecture
